# From home to the screen: How parental rejection fuels cyberbullying in college students

**DOI:** 10.1371/journal.pone.0323124

**Published:** 2025-05-27

**Authors:** Shuaijie Lan, Yangan Wang, Jiaxu Zhao, Xujing Hou, Chao Li

**Affiliations:** 1 School of Psychology, Northeast Normal University, Changchun, Jilin, China; 2 Changqing No. 1 High School, Changqing District, Jinan, Shandong, China; 3 Student Psychological Development Guidance Center, BeiHua University, Jilin, Jilin, China.; University of Cambridge, UNITED KINGDOM OF GREAT BRITAIN AND NORTHERN IRELAND

## Abstract

Previous research has highlighted the impact of family environment on college students’ cyberbullying behavior, yet the role of parenting styles, particularly negative ones, remains underexplored. This study, grounded in the interpersonal acceptance-rejection theory and social information processing model, investigates how parental rejection influences cyberbullying behavior among college students through cognitive and emotional mechanisms. We surveyed 1,567 college students (620 males, 947 females; average age: 19.34 ± 1.24 years) from several universities in Shandong and Jilin provinces, China. Participants completed questionnaires assessing cyberbullying, parental rejection, empathy, and moral disengagement. The findings reveal that 456 individuals (29.1%) had engaged in at least one instance of cyberbullying behavior, including 180 males and 276 females. Subsequently, an investigation into the cyberbullying behaviors of these individuals revealed that: (1) parental rejection is a significant predictor of cyberbullying behavior; (2) empathy and moral disengagement serve as partial mediators in the relationship between parental rejection and cyberbullying; (3) both empathy and moral disengagement act as sequential mediators in this relationship. These results underscore the importance of empathy and moral disengagement in understanding the link between parental rejection and cyberbullying among college students, offering a new theoretical perspective for future interventions.

## 1 Introduction

While the information age has brought unprecedented convenience through internet technologies, its dark side—such as cyberbullying—has also come to light. Cyberbullying consists of the ongoing transmission of hostile or aggressive messages by individuals or groups via electronic devices, leading to psychological distress for the victims [1]. In the research on cyberbullying, the issues faced by adolescents initially received widespread attention, with related studies primarily focusing on this demographic for a considerable period [2]. However, recent findings indicate that the problem of cyberbullying among college students may be even more severe.

On one hand, study have found that cyberbullying behaviors in adolescents tend to persist, with those involved in cyberbullying during their teenage years often continuing such behaviors into their college years [3].On the other hand, college students spend significantly more time online than adolescents [4]. Surveys suggested that college students spend average about 10 hours per day on the internet [5], which markedly increases their risk of engaging in cyberbullying behaviors [6]. The impact of cyberbullying on victims is profound, potentially leading to psychological symptoms such as anxiety and depression [7,8], which can interfere with their daily lives and studies, and in severe cases, may even lead to tragic outcomes such as suicide [9]. Given the prevalence of cyberbullying among college students and its serious consequences, investigating its influencing factors is not only of significant academic value but also holds strong practical significance.

The role of family environment has attracted researchers’ attention in studying the influencing factors of cyberbullying among college students. Existing studies have found that college students who have left-behind experiences or come from lower socioeconomic backgrounds are more likely to engage in cyberbullying [10,11]. However, there is limited research exploring the impact of parenting styles on cyberbullying behaviors in this demographic. As the first teachers of their children, parents’ behavioral patterns towards their offspring often have profound effects on their development. Study indicated that children who perceive parental acceptance typically exhibit less hostility, while those who perceive parental rejection tend to display more aggression, an effect that persists even into adulthood [12]. Thus, parental rejection may be an important factor influencing cyberbullying among college students, yet relevant research remains insufficient.

Additionally, although few studies have explored the relationship between parenting styles and cyberbullying among college students, mostly considered cognitive or emotional mechanisms in isolation, failing to integrate both [13,14], which are insufficient to comprehensively explain the complete occurrence pathway of cyberbullying. Therefore, further investigation into the role of parenting styles in cyberbullying among college students is needed. This study, grounded in the interpersonal acceptance-rejection theory and incorporating relevant perspectives from the social information processing model, explores the emotional and cognitive mechanisms through which parental rejection influences cyberbullying. It provides new insights into understanding the causes of cyberbullying among college students.

### 1.1 Parental rejection and cyberbullying

Parental rejection is a negative parenting style characterized by a lack of warmth, patience, care, and support during a child’s development [15]. According to the Interpersonal Acceptance-Rejection (IPAR) Theory, as the most influential figures in a child’s development, parents play a crucial role in shaping how the child perceives themselves and others [16]. Compared to parental acceptance, such as effective communication and warmth, which promote a positive worldview, parental rejection often has a profound negative impact on a child’s worldview [17]. Rejection from parents can lead the child to internalize feelings of being unworthy of love, view interpersonal relationships as cold and indifferent, and perceive the world as hostile and dangerous. This negative worldview promotes the formation of an antagonistic cognitive schema, which may persist into the college years [12]. Under the influence of this negative cognitive framework, college students who experienced parental rejection tend to interpret social information negatively, perceiving it as hostility and provocation directed at themselves, which can lead to aggressive responses [18].

Previous studies have also shown that youth who frequently experienced parental rejection during childhood are more likely to exhibit aggressive behaviors, including school bullying, emotional abuse, and physical aggression [19–21]. As far as we know, while no study has directly investigated the relationship between parental rejection and cyberbullying among college students, existing empirical evidence from adolescent populations in both the United States and China indicates that parental rejection is linked to a higher likelihood of engaging in cyberbullying [22,23].

Given the evidence that cyberbullying exhibits longitudinal continuity [3], parental rejection may serve as a key predictor of such behaviors in college students.

### 1.2 The mediating role of empathy

In the context of cyberbullying, the Social Information Processing (SIP) Model is commonly used to understand how individuals interpret and respond to online interactions. According to SIP Model, the behaviors that individuals exhibit in social interactions are the result of their interpretation of the current environmental information [24]. This process not only relies on the cognitive frameworks that individuals have developed over time but also on how they interpret the emotional signals of others, which in turn influences their understanding of the social information conveyed by others. The ability to understand, feel, and share another person’s emotions is known as empathy [25]. Previous study had shown that college students who lack emotional cue recognition and empathy often fail to “feel the pain of others,” leading to misinterpretations of social information. These misinterpretations, in turn, increase the likelihood of pursuing harmful interpersonal goals, which may result in aggressive behavior [26]. In contrast, individuals with higher empathy can think from others’ perspectives and accurately identify the emotional information conveyed by others, thus exhibiting less aggressive behavior towards others [27].

In the process of individual development, empathy is not a fixed trait but is influenced by various factors, including parenting styles [28,29]. When parents are able to understand and respond to their children’s emotional needs—such as by listening to and affirming their feelings and providing a warm and supportive environment—it significantly promotes the development of empathy. Previous research has found that emotionally warm parenting styles contribute to the development of empathy in college students [30]. A longitudinal study spanning thirty years further confirmed that children who received emotional acceptance and warmth from their parents were more likely to maintain higher levels of empathy well into adulthood [31]. Conversely, when parents are emotionally distant or reject their children, it difficult for children to understand emotional cues from others, potentially resulting in lower levels of empathy. Recent empirical studies have confirmed this viewpoint, showing that college students with lower levels of empathy are more likely to feel rejected by their parents [32].

Based on the evidence presented, it can be inferred that empathy may play a mediating role in the relationship between parental rejection and college students’ cyberbullying.

### 1.3 The mediating role of moral disengagement

Based on the SIP Model, individuals interpret and evaluate their behavioral goals before engaging in social behavior, and only when they deem the goal reasonable do they proceed with the corresponding social action [24]. As mentioned earlier, a study has found that college students who experience prolonged parental rejection are more likely to adopt aggression as a behavioral goal [21]. However, engaging in aggressive behavior is unethical, and individuals who partake in such actions may experience painful emotions such as guilt. In these circumstances, individuals need a cognitive mechanism to rationalize the target of aggression, reducing inner distress. Moral disengagement serves this purpose. Moral disengagement is defined as a set of cognitive mechanisms that allow individuals to justify their unethical behavior by employing strategies such as cognitive restructuring and externalizing responsibility, thereby reducing guilt and other negative emotions [33]. The anonymity and virtual nature of the online environment provide fertile ground for moral disengagement, making it easier for individuals to attribute responsibility to external factors and exonerate themselves for their aggressive behavior [34].

It is noteworthy that the family environment—especially parenting styles—plays a crucial role in shaping children’s levels of moral disengagement. For instance, research by Pentima et al. discovered a negative correlation between warm and supportive parenting styles in childhood and the levels of moral disengagement in adulthood [35]. Similarly, research by Vaden et al. demonstrated that parental emotional warmth contributes to promoting moral development in college students, leading to higher levels of moral identity [36]. Conversely, parental rejection tends to be positively correlated with children’s levels of moral disengagement [37]. These patterns are observed not only in children but also apply to adolescents and college students, indicating that this relationship is consistent across different developmental stages [38,39].

Therefore, moral disengagement may play a mediating role in the impact of parental rejection on college students’ cyberbullying.

### 1.4 The serial mediating role of empathy and moral disengagement

According to the SIP model, individuals’ social behaviors undergo four stages: information decoding, target setting, target evaluation, and behavior execution [24]. College students who have experienced parental rejection for a long time often exhibit lower levels of empathy [32]. This deficiency leads them to interpret social information conveyed by others in social networks as hostility and provocation signals [27]. Consequently, they are more likely to set aggressive targets in their social activities [40].

Individuals who lack empathy create aggressive targets that require cognitive restructuring through moral disengagement to justify their actions, enabling them to translate their intentions into actual behavior [41]. This results in their more frequent use of moral disengagement mechanisms during the target evaluation stage. Previous study has demonstrated that college students with lower levels of empathy tend to exhibit higher levels of moral disengagement [42]. Longitudinal research further supports this, demonstrating that empathy levels can significantly predict individuals’ moral disengagement levels [43]. Furthermore, lower empathy levels and higher moral disengagement levels are closely associated with a range of problematic behaviors, including school bullying and cyberbullying [44,45].

Given this evidence, we posit that empathy and moral disengagement are likely to function as the affective and cognitive mechanisms, respectively, through which parental rejection influences college students’ cyberbullying.

### 1.5 The present study

In summary, parental rejection is likely to influence cyberbullying among college students, with empathy and moral disengagement potentially serving as mediators in this relationship. However, the specific relationship between parental rejection and cyberbullying among college students remains unclear, and the mechanisms underlying this relationship have not been thoroughly explored. Therefore, this study, grounded in the IPAR Theory and the SIP Model, proposes the following four hypotheses:

Hypothesis 1: There is a significant positive correlation between parental rejection and cyberbullying behaviors among college students.

Hypothesis 2: Empathy plays a mediating role between parental rejection and cyberbullying behaviors among college students.

Hypothesis 3: Moral disengagement plays a mediating role between parental rejection and cyberbullying behaviors among college students.

Hypothesis 4: Empathy and moral disengagement serve as serial mediators between parental rejection and cyberbullying behaviors among college students.

Through the exploration of these relationships, this study systematically reveals the cognitive and emotional mechanisms through which negative parenting styles, specifically parental rejection, influence college students’ cyberbullying behavior. Our study extends the IPAR Acceptance-Rejection Theory to the domain of cyberbullying among college students, thus broadening the scope of this theory. Furthermore, this study considers both the emotional and cognitive mechanisms through which parenting styles influence cyberbullying among college students, providing a more comprehensive understanding of cyberbullying behavior in this demographic. Additionally, by integrating the TPAR Theory and the SIP Model, this study not only provides a new perspective for intervening in cyberbullying among college students but also suggests specific and actionable measures for intervention.

## 2 Materials and methods

### 2.1 Participants

Considering the homogeneous characteristics of the university student population involved in cyberbullying [46], this study employed a simple random sampling method, randomly selecting samples from several universities in Shandong and Jilin provinces for questionnaire survey. Data collection occurred between June 3 and June 30, 2024, with 1,655 undergraduates voluntarily completing an 85-item online questionnaire via the Questionnaire Star platform (www.wjx.cn). After excluding 87 invalid questionnaires (5.32%), a total of 1,568 valid responses were obtained (validity rate of 94.68%). The average age of participants was 19.34 years (standard deviation = 1.24), with 620 males (40.34%) and 947 females (60.44%).

### 2.2 Ethics statement

All participants read the informed consent form prior to completing the questionnaire, which clearly stated: (1) they could withdraw at any time without reason and would not be affected; (2) data would be stored anonymously and used solely for academic research; (3) a reward of 2 RMB (approximately 0.28 USD) would be provided upon completion of the questionnaire. This study was approved by the Academic Ethics Committee of the College of Psychology at Northeast Normal University (approval number: 2024-PSY-015).

### 2.3 Measures

#### 2.3.1 Cyberbullying.

The Cyberbullying Scale, developed by Erdur and Kavsut [47] and subsequently refined by Zhou et al. [48], is widely recognized for its reliability and validity in investigating cyberbullying among college students. This scale comprises 18 items to evaluate participants’ online bullying behaviors, including statements such as “Spreading rumors about someone on the internet.” Participants rated their experiences using a 4-point Likert scale that assesses the frequency of cyberbullying incidents over the past three months, with response options ranging from 1 (“never”) to 4 (“more than five times”). A total score exceeding 18 on the Cyberbullying Scale signifies at least one instance of cyberbullying behavior, with higher scores reflecting more frequent engagement in such behaviors. In this study, the Cyberbullying Scale exhibited a Cronbach’s α of 0.807. The confirmatory factor analysis (CFA) revealed marginally acceptable fit indices for the unidimensional model (CFI = 0.838, TLI = 0.817, RMSEA = 0.060, SRMR = 0.053), possibly attributable to the influence of social desirability bias [49].

#### 2.3.2 Parental rejection.

Parental rejection was evaluated using the 6-item Parental Rejection subscale from the Chinese version of the Short-Form Egna Minnen av Barndoms Uppfostran (s-EMBU-C) [50]. This scale primarily assesses parental rejection behavior by asking individuals to recall how their parents treated them during their developmental years. One representative item is: “Even minor mistakes will lead to punishment from my parents.” Participants evaluated each item on a 4-point scale, where 1 represented ‘rarely’ and 4 represented ‘almost always.’ The results of this study indicated a Cronbach’s α of 0.918 for the scale. Confirmatory factor analysis (CFA) demonstrated good construct validity of the scale (CFI = 0.955, TLI = 0.945, RMSEA = 0.069, SRMR = 0.035).

#### 2.3.3 Moral disengagement.

The Chinese version of the Moral Disengagement Scale consists of 26 items [51], with a representative item: “Talking about others behind their backs is just a joke.” Participants responded on a 5-point scale, where one indicated “strongly disagree” and five indicated “strongly agree.” Scores for the 26 items were summed, with higher scores indicating a greater moral disengagement. In this study, the Cronbach’s α for the scale was 0.941. The confirmatory factor analysis (CFA) indicated good structural validity for the scale (CFI = 0.945, TLI = 0.941, RMSEA = 0.044, SRMR = 0.036).

#### 2.3.4 Empathy.

The Basic Empathy Scale was developed by Joliffe [52]. This scale uses a 5-point Likert scale ranging from 1 (strongly disagree) to 5 (strongly agree). One representative item is: “I can usually understand how someone feels when they are down.” After reverse scoring certain items, the overall score of the scale was computed, where increased scores reflect greater levels of empathy. The Cronbach’s α coefficient for the scale in this study was found to be 0.934. Confirmatory factor analysis (CFA) demonstrated satisfactory construct validity of the scale (CFI = 0.965, TLI ＝ 0.961, RMSEA＝0.042, SRMR ＝0.032).

### 2.4 Procedure and data analyses

First, Monte Carlo simulations were employed to analyze the statistical power of the sample size [53]. Following that, we calculated the incidence of cyberbullying and analyzed gender differences. Given that all participants’ data were collected using self-report scales, there is a potential risk of common method bias. To mitigate this concern, we applied the Harman single-factor method for testing. Afterward, we conducted multicollinearity diagnostics on the regression model. Subsequently, we performed both descriptive and correlational analyses to explore the relationships among the variables. Finally, to further investigate the connections between parental rejection, cyberbullying, empathy, and moral disengagement, we conducted an in-depth analysis using SPSS 26.0 and PROCESS 3.5 [54].

## 3 Results

### 3.1 The occurrence of cyberbullying among college students and analysis of demographic variables

An analysis of the total scores on the cyberbullying scale revealed that 456 individuals (29.1%) had engaged in at least one instance of cyberbullying behavior, including 180 males and 276 females. The results of an independent samples t-test revealed no significant differences in cyberbullying behavior between genders (*p* = 0.180).

### 3.2 Power analysis

A post-hoc Monte Carlo simulation was conducted to estimate the model’s power [53]: setting the sample size (N = 456) with 1000 Power Analysis Replications (5000 Monte Carlo Draws for Replications) and a confidence level of 95%, the tool returned the power of 1 for the first indirect path (parental rejection - empathy - cyberbullying) and of 0.98 for the second indirect path parental rejection – moral disengagement - cyberbullying). The power for the third indirect path (parental rejection – empathy – cyberbullying – moral disengagement) was also 0.98. Therefore, the power of the model was adequate.

### 3.3 Test of common method biases and collinearity diagnostics

Specific methodological errors may occur because the statistical data rely exclusively on the questionnaires completed according to participants’ subjective intentions. We employed Harman’s single-factor analysis to mitigate potential standard method biases inherent to this research approach. The analysis showed that there were sixteen factors with eigenvalues greater than one, and the leading factor accounted for 20.8% of the variance. This figure is below the critical threshold of 40%, indicating that our study is likely within an acceptable range regarding the influence of common method biases [55].

Collinearity diagnostics showed the variance inflation factor (VIF) for parental rejection, empathy, and moral disengagement was 1.179,1.182,1.172, respectively, which was below the threshold of 5 [56]. Thus, the multi-collinearity might not affect our estimates.

### 3.4 Descriptive statistics

Table 1 presents the mean, standard deviation, and correlation matrix for each research variable. Additionally, we conducted skewness and kurtosis tests on the four variables of the results equation model. The findings suggested that the data generally met the skewness and kurtosis criteria necessary for structural equation modeling [57].

**Table 1:** Descriptive statistics.

Variables	M	SD	1	2	3	4	Skewness	Kurtosis
1.Cyberbullying	20.599	3.053	–				2.488	5.364
2. Parental rejection	24.070	8.729	0.277^**^	–			0.903	0.062
3. Empathy	77.476	17.110	-0.365^**^	-0. 322^**^	–		-1.440	1.943
4. Moral disengagement	50.836	19.228	0.355^**^	0.310^**^	-0.314^**^	–	1.668	3.286

**Note.** **p < .01;

Furthermore, the results indicated that empathy was negatively related to cyberbullying, parental rejection, and moral disengagement, respectively, while cyberbullying, parental rejection, and moral disengagement were positively correlated with each other.

### 3.5 Testing for the proposed mediating model

We employed Model 6 of the PROCESS macro with 5,000 bootstrap samples to investigate the mediating roles of empathy and moral disengagement, with gender included as a covariate in the analysis. To minimize Type, I error due to the data distribution, the unstandardized regression coefficient was used [58]. The findings from our analysis are outlined in Table 2. The findings demonstrate that parental rejection directly predicts moral disengagement and exerts a significant positive effect on cyberbullying. Conversely, parental rejection significantly negatively impacts empathy. Additionally, the findings indicate that empathy negatively influences both moral disengagement and cyberbullying. Furthermore, moral disengagement is found to predict cyberbullying behavior positively.

**Table 2:** Regression analysis.

Model		Fit Index			Significance	
**Outcome Variable**	**Predictor Variable**	***R*** ^***2***^	***F***	**β**	**t**	**95%CI**
Empathy	Sex	0.113	28.867	-3.405	-2.201*	[-6.446,-0.356]
	Parental rejection			-0.630	-7.259***	[-0.800,-0.459]
Moral disengagement	Sex	0.148	26.124	1.132	0.660	[-2.239,4.503]
	Empathy			-0.265	-5.108***	[-0.369,-0.167]
	Parental rejection			0.515	5.095***	[0.316,0.713]
Cyberbullying	Sex	0.211	30.092	0.155	0.589	[-0.361,0.671]
	Empathy			-0.044	-5.429***	[-0.060,-0.028]
	Moral disengagement			0.038	5.256***	[0.024,0.052]
	Parental rejection			0.043	2.704**	[0.012,0.074]

**Note.** **p < .01, ***p < .001; β, unstandardized regression coefficients

**Fig 1:**
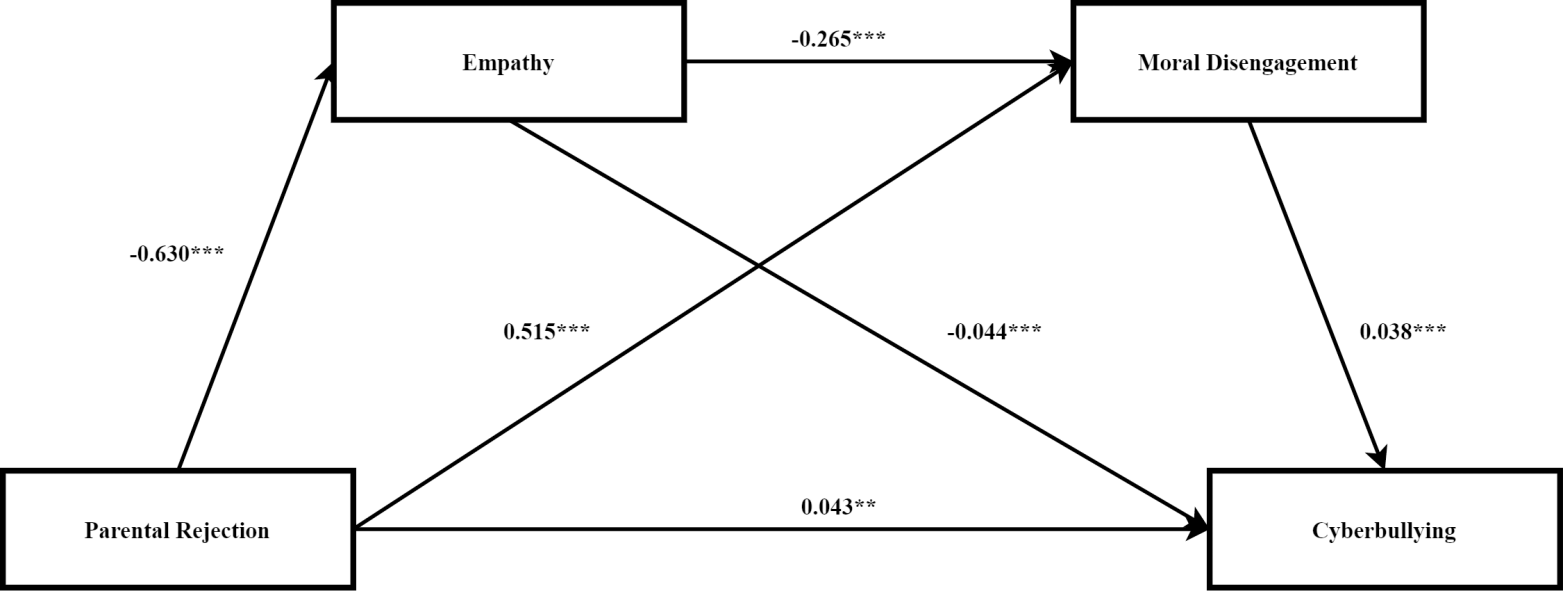
Roadmap of the influence of parental rejection on cyberbullying. Note. ** *p*  < 0.01, *** *p*  < 0.001.

The results indicate that the total effect of parental rejection on cyberbullying was significant (β = 0.097). Furthermore, parental rejection significantly influenced cyberbullying indirectly through empathy (β = 0.028), accounting for 28.87% of the total effect. The indirect influence of parental rejection on cyberbullying through moral disengagement was found to be significant (β = 0.019), accounting for 19.59% of the overall effect. The serial mediation effect was also significant (β = 0.006), accounting for 6.19% of the total effect, supporting our model hypothesis. The comprehensive findings can be found in Table 3, and Fig 1 illustrates the connection between parental rejection and cyberbullying.

**Table 3:** Total, direct, and indirect effects.

	β	Boot SE	LLCI/ULCI (95%)		Relative Value
**LLCI**	**ULCI**	
PR → EP → CB	0.028	0.009	0.012	0.046	28.9%
PR → MD → CB	0.019	0.006	0.009	0.032	19.6%
PR → EP → MD → CB	0.006	0.003	0.002	0.013	6.2%
Direct effect	0.043	0.016	0.011	0.074	44.3%
Total indirect effect	0.054	0.011	0.033	0.077	55.7%
Total effect	0.097	0.016	0.066	0.128	

**Note.** PR, parental rejection; EP, empathy; CB, cyberbullying; MD, moral disengagement; LLCI, lower limit confidence interval; ULCI, upper limit interval; β, unstandardized regression coefficients

## 4 Discussion

### 4.1 The effects of parental rejection on cyberbullying

Research findings indicated that parental rejection behaviors significantly positively predict online bullying behaviors among college students (accounting for 44.3% of the total effect), supporting Hypothesis 1. This discovery is consistent with previous studies on the relationship between parental rejection and adolescent online bullying [22,23], suggesting that parental rejection not only affects adolescents’ online bullying behaviors but also that this impact persists as individuals enter college.

According to the IAPR Theory, parental rejection influences children’s cognitive and emotional mechanisms, which in turn shape their behavioral patterns in adulthood [16]. College students who grow up in environments marked by parental rejection and denial often experience a weakening of their self-confidence, leading them to feel unworthy of love and lacking a sense of self-worth. This negative self-concept gradually permeates their worldview, causing them to perceive the world as hostile and negative. This cognitive framework persists into adulthood [12]. The SIP Model suggests that an individual’s social behavior is shaped by their interpretation of the surrounding environment [24]. For college students who have experienced parental rejection, their negative cognitive patterns lead them to perceive others as threatening and unsafe, making them overly alert and sensitive [18]. Consequently, these individuals are more likely to interpret social cues in online environments as “signals of attack,” which can result in reactions such as cyberbullying.

Moreover, college students who have been repeatedly rejected by their parents often have unmet emotional needs. Over time, these unmet needs can lead to the development of anger and resentment, resulting in the accumulation of destructive emotions [12]. In this emotional state, they become irritable and emotionally unstable [59]. Additionally, parental rejection hinders their ability to learn effective emotion regulation strategies [60]. As a result, these individuals are more likely to resort to aggressive behaviors as a means of coping with negative emotions. Furthermore, the anonymity of the online environment offers them an unrestricted outlet for emotional venting, which can lead to more frequent expressions of negative emotions through cyberbullying behaviors [61].

### 4.2 The mediating role of empathy

Research findings indicate that empathy partially mediates the relationship between parental rejection and cyberbullying among college students (accounting for 28.9% of the total effect and 51.6% of the total indirect effect), thereby supporting Hypothesis 2.

According to the Interpersonal Acceptance-Rejection Theory, parents, as the primary attachment figures for children, play a crucial role in the development of children’s emotional competencies through positive emotional responses [16]. A warm and positive parenting style enables parents to respond promptly to the emotional signals conveyed by their children, fostering emotional resonance and helping them gradually acquire empathy as they grow [62]. As empathy develops, college students become better equipped to understand the emotional signals of others, allowing them to accurately interpret social information in online environments and consequently reduce instances of cyberbullying [30].

Conversely, individuals who experience prolonged parental rejection face a dual risk in the development of empathy. On one hand, the lack of positive emotional interactions prevents them from learning to understand and evaluate the emotional signals of others, resulting in generally lower social skills [63]. On the other hand, rejection within sustained intimate relationships leads to emotional withdrawal, making them reluctant to express their feelings or engage in emotional communication with others [64], which further hampers the development of their empathic abilities. Furthermore, the difficulties in interpreting others’ emotional signals, combined with the lack of social feedback in online environments, increase the likelihood that they will misinterpret social signals from others as hostility, subsequently leading to more aggressive behaviors.

### 4.3 The mediating role of moral disengagement

Research findings indicate that moral disengagement plays a partial mediating role (accounting for 19.6% of the total effect and 35.2% of the total indirect effect) between parental rejection and cyberbullying among college students, thereby validating Hypothesis 3.

One manifestation of parental rejection is the neglect of children’s physical, psychological, and social needs [65], essentially failing to fulfill their parenting responsibilities. However, such parents often remain unaware of their behavioral issues and frequently use moral disengagement mechanisms to justify their actions, attributing responsibility to the child and believing that everything is the child’s fault.

According to social learning theory, children acquire this cognitive pattern by observing and imitating their parents’ behaviors [66]. Consequently, college students raised in such an environment may gradually adopt similar behavioral patterns, leading to a decline in their moral standards and an increased likelihood of using moral disengagement strategies to rationalize negative behaviors. Within the framework of social information processing models, once they interpret social information as hostile, they may decide to engage in online bullying behaviors. Even when they recognize that such actions are unethical, they still seek reasons to justify their behavior, attempting to rationalize it.

### 4.4 Exploring the serial mediation of empathy and moral disengagement in the relationship between parental rejection and cyberbullying in college students

The results of this study indicate that parental rejection initially affects college students’ empathy, which subsequently influences their moral disengagement, ultimately contributing to the occurrence of cyberbullying behavior (accounting for 6.2% of the total effect and 11.11% of the total indirect effect), thereby validating Hypothesis 4.

Based on the SIP model, individuals in an online environment must first process social information, which includes understanding the events that occur and analyzing their causes [24]. Parenting styles characterized by parental rejection may lead college students to develop negative personality traits, resulting in hostile perceptions of social information [12]. Furthermore, a lack of empathy prevents them from accurately interpreting others’ emotional signals and incorporating these signals into effective processing of social information, making them more likely to misinterpret these signals as provocations [18]. This cognitive bias further encourages individuals to perceive cyberbullying behavior as a target. However, they also recognize that such behavior is immoral, which leads them to employ mechanisms of moral disengagement to justify their actions, thereby rationalizing their behavior and alleviating the impact of negative emotions [33]. Therefore, parental rejection significantly impacts college students’ cyberbullying behavior through both cognitive and emotional mechanisms.

## 5. Conclusions

In conclusion, this study indicates that parental rejection can positively predict cyberbullying behavior among college students. Furthermore, the study employs the social information processing model to elucidate the mediating role of empathy and moral disengagement as emotional and cognitive mechanisms between parental rejection and cyberbullying. Therefore, to more effectively prevent and address cyberbullying among university students, it is essential not only to strengthen school interventions but also to pay greater attention to the influence of the family environment. Parents can strengthen emotional education in the early years of their children and help them establish proper moral understanding, thereby effectively reducing the risk of encountering cyberbullying when they enter university.

## 6 Implications

This study offers a new theoretical perspective on the emergence and development of cyberbullying behavior among college students by integrating the social information processing model with the interpersonal acceptance-rejection theory, specifically focusing on parental rejection. Furthermore, this study reveals that parental rejection not only directly influences cyberbullying behavior but also indirectly affects it through empathy and moral disengagement, highlighting its practical significance.

While most previous research has focused on schools as critical settings for addressing cyberbullying among college students and advocating for school-based interventions, this study aims to raise parents’ awareness of their role in this issue. By examining how parental rejection influences college students’ cyberbullying behavior, parents can better understand the significant impact of their parenting styles on their children’s engagement in cyberbullying—an influence that may persist into adulthood. Moreover, the study offers parents educational concepts and coping strategies to better support and guide their children. For example, when conflicts arise between parents and children, parents should avoid solely blaming their children. Instead, they should first stabilize their emotions and engage in constructive communication, fostering emotional connections with their children. This approach allows children to experience the warmth of family, gradually reshaping their hostile perceptions of the world. Additionally, parents can encourage their children to develop empathy by prompting them to consider the feelings of others, such as by asking, “How do you think your friend would feel if they heard you say that?” This process can help children enhance their ability to empathize with others, ultimately reducing the cyberbullying they experience after entering college.

## 7. Limitations

Despite the current study’s progress in exploring the antecedents and mechanisms of cyberbullying behaviors among college students, several limitations remain that future research needs to address. Firstly, this study employed a cross-sectional design, lacking longitudinal data for support. Therefore, the reliability of the conclusions needs to be further validated through longitudinal research. Second, this study examines participants’ experiences with cyberbullying through self-reports. Due to the potential influence of social desirability bias, many college students may hesitate to answer questions about cyberbullying truthfully, as they may be reluctant to label themselves as perpetrators [67]. Although we emphasized anonymity during the testing process and randomized the question in order to minimize the impact of social desirability bias, we cannot fully guarantee that participants responded honestly about their experiences with cyberbullying. Thus, future research should adopt methods that can enhance the reliability of cyberbullying reporting. Techniques such as social media analysis or digital footprint data can provide a more objective assessment of cyberbullying behaviors. Additionally, the sample in this study was primarily drawn from undergraduate students in Shandong and Jilin provinces in China. While this sample may somewhat represent the broader Chinese university student population, the extent to which these findings can be generalized to university students from other countries remains uncertain due to cultural differences. This limitation may lead to insufficient representativeness and restrict the generalizability of the conclusions. Therefore, future research should consider expanding the geographical scope of the sample to include college students from diverse regions and cultural backgrounds to enhance the external validity and applicability of the findings.

## References

[1] Tokunaga RS. Following you home from school: A critical review and synthesis of research on cyberbullying victimization. Computers in Human Behavior. 2010;26(3):277–87. doi: 10.1016/j.chb.2009.11.014

[2] Watts LK, Wagner J, Velasquez B, Behrens PI. Cyberbullying in higher education: A literature review. Computers in Human Behavior. 2017;69:268–74. doi: 10.1016/j.chb.2016.12.038

[3] Chapell M, Hasselman S, Kitchin T, Lomon S, Maclver K, Sarullo P. Bullying in elementary school, high school, and college. Adolescence. 2006;41:633–48. 17240771

[4] Salleh SM, Hamzah SFM, Ali NM, Yusof HSM, Mohammed NH, Nor RM, et al. The association between age and time spent on online activity among university students. International Journal of Academic Research in Business and Social Sciences. 2023;13: Pages 1480-–1488. doi: 10.6007/IJARBSS/v13-i12/20049

[5] Baroni A, Feder MA, Castellanos FX, Li J, Shatkin J. Internet use 101 in college: Do undergraduates want to learn healthier internet use?. Public Health Pract (Oxf). 2023;6:100411. doi: 10.1016/j.puhip.2023.100411 37576526 PMC10413188

[6] Hinduja S, Patchin JW. Cyberbullying: An exploratory analysis of factors related to offending and victimization. Deviant Behavior. 2008;29(2):129–56. doi: 10.1080/01639620701457816

[7] Lam TN, Jensen DB, Hovey JD, Roley-Roberts ME. College students and cyberbullying: How social media use affects social anxiety and social comparison. Heliyon. 2022;8(12):e12556. doi: 10.1016/j.heliyon.2022.e12556 36619438 PMC9816968

[8] Zhang D, Huebner ES, Tian L. Longitudinal associations among neuroticism, depression, and cyberbullying in early adolescents. Computers in Human Behavior. 2020;112:106475. doi: 10.1016/j.chb.2020.106475

[9] Tabares ASG, Restrepo JE, Zapata-Lesmes G. The effect of bullying and cyberbullying on predicting suicide risk in adolescent females: The mediating role of depression. Psychiatry Res. 2024;337:115968. doi: 10.1016/j.psychres.2024.115968 38820653

[10] Wang H, Wu S, Wang W, Xiao Y. Left-behind experiences and cyberbullying behavior in Chinese college students: The mediation of sense of security and the moderation of gender. Behav Sci (Basel). 2023;13(12):1001. doi: 10.3390/bs13121001 38131857 PMC10740690

[11] Sun M, Ma Z, Xu B, Chen C, Chen Q-W, Wang D. Prevalence of cyberbullying involvement and its association with clinical correlates among Chinese college students. J Affect Disord. 2024;367:374–81. doi: 10.1016/j.jad.2024.08.198 39236886

[12] Khaleque A, Rohner RP. Pancultural associations between perceived parental acceptance and psychological adjustment of children and adults. Journal of Cross-Cultural Psychology. 2011;43(5):784–800. doi: 10.1177/0022022111406120

[13] Sun Z, Ding W, Chu X, Xie R, Li J, Jiang M, et al. The relationship between perceived childhood harsh parental discipline and cyberbullying among college students: A moderated mediation model. J Adult Dev. 2022;30(4):321–33. doi: 10.1007/s10804-022-09432-5

[14] Chen L, Wang Y, Yang H, Sun X. Emotional warmth and cyberbullying perpetration attitudes in college students: Mediation of trait gratitude and empathy. PLoS One. 2020;15(7):e0235477. doi: 10.1371/journal.pone.0235477 32663843 PMC7360375

[15] Rohner RP, Khaleque A, Cournoyer DE. Parental acceptance‐rejection: Theory, methods, cross‐cultural evidence, and implications. Ethos. 2005;33(3):299–334. doi: 10.1525/eth.2005.33.3.299

[16] Rohner RP, Lansford JE. Deep structure of the human affectional system: Introduction to interpersonal acceptance–rejection theory. J of Family Theo & Revie. 2017;9(4):426–40. doi: 10.1111/jftr.12219

[17] Khaleque A. Perceived parental hostility and aggression, and children’s psychological maladjustment, and negative personality dispositions: A meta-analysis. J Child Fam Stud. 2016;26(4):977–88. doi: 10.1007/s10826-016-0637-9

[18] Khaleque A, Uddin MK, Hossain KN, Siddique MdN-E-A, Shirin A. Perceived parental acceptance–rejection in childhood predict psychological adjustment and rejection sensitivity in adulthood. Psychol Stud. 2019;64(4):447–54. doi: 10.1007/s12646-019-00508-z

[19] Sabah A, Aljaberi MA, Lin C-Y, Chen H-P. The associations between sibling victimization, sibling bullying, parental acceptance-rejection, and school bullying. Int J Environ Res Public Health. 2022;19(23):16346. doi: 10.3390/ijerph192316346 36498416 PMC9739229

[20] Tussey BE, Tyler KA, Simons LG. Poor parenting, attachment style, and dating violence perpetration among college students. J Interpers Violence. 2021;36(5–6):2097–116. doi: 10.1177/0886260518760017 29475423

[21] Zhu L, Huang M, Fang Z, Tong J, Pan Z, Hua L, et al. Exploring the relationship between aggressive behavior, family parenting styles, and self-esteem among only-child college students in China: A cross-sectional study. Psychol Res Behav Manag. 2025;18:435–48. doi: 10.2147/PRBM.S505802 40026338 PMC11872094

[22] Hinduja S, Patchin JW. Bullying and cyberbullying offending among US youth: The influence of six parenting dimensions. J Child Fam Stud. 2022;31(5):1454–73. doi: 10.1007/s10826-021-02208-7

[23] Wang J, Zheng S, Zhang M, Kedi Zhao. Parenting styles and adolescents’ cyberbullying: The indirect effect of attitude towards human dignity. J Child Fam Stud. 2025;34(1):270–81. doi: 10.1007/s10826-024-02971-3

[24] Lemerise EA, Arsenio WF. An integrated model of emotion processes and cognition in social information processing. Child Dev. 2000;71(1):107–18. doi: 10.1111/1467-8624.00124 10836564

[25] Håkansson Eklund J, Summer Meranius M. Toward a consensus on the nature of empathy: A review of reviews. Patient Educ Couns. 2021;104(2):300–7. doi: 10.1016/j.pec.2020.08.022 32888755

[26] Loudin JL, Loukas A, Robinson S. Relational aggression in college students: Examining the roles of social anxiety and empathy. Aggressive Behavior. 2003;29(5):430–9. doi: 10.1002/ab.10039

[27] Qiu Y, Sun Q, Wu B, Li F. Is high exposure to antisocial media content associated with increased participation in malicious online trolling? Exploring the moderated mediation model of hostile attribution bias and empathy. BMC Psychol. 2024;12(1):401. doi: 10.1186/s40359-024-01898-0 39030650 PMC11264487

[28] Streit C, Carlo G, Killoren SE. Family support, respect, and empathy as correlates of U.S. Latino/Latina college students’ prosocial behaviors toward different recipients. Journal of Social and Personal Relationships. 2020;37(5):1513–33. doi: 10.1177/0265407520903805

[29] Maiya S, Streit C, Kline GC. Sibling relationship qualities and prosocial behaviors in Asian Indian college students: Intervening roles of family respect values and empathy. Journal of Social and Personal Relationships. 2024. doi: 10.1177/02654075241298713

[30] Chen L, Wang Y, Yang H, Sun X. Emotional warmth and cyberbullying perpetration attitudes in college students: Mediation of trait gratitude and empathy. PLoS One. 2020;15(7):e0235477. doi: 10.1371/journal.pone.0235477 32663843 PMC7360375

[31] Hintsanen M, Gluschkoff K, Dobewall H, Cloninger CR, Keltner D, Saarinen A, et al. Parent–child-relationship quality predicts offspring dispositional compassion in adulthood: A prospective follow-up study over three decades. Dev Psychol. 2019;55(1):216–25. doi: 10.1037/dev0000633 30431291

[32] Li C-Q, Ma Q, Liu Y-Y, Jing K-J. Are parental rearing patterns and learning burnout correlated with empathy amongst undergraduate nursing students?. Int J Nurs Sci. 2018;5(4):409–13. doi: 10.1016/j.ijnss.2018.07.005 31406856 PMC6626275

[33] Gini G, Pozzoli T, Hymel S. Moral disengagement among children and youth: A meta-analytic review of links to aggressive behavior. Aggress Behav. 2014;40(1):56–68. doi: 10.1002/ab.21502 24037754

[34] Chan TKH, Cheung CMK, Benbasat I, Xiao B, Lee ZWY. Bystanders join in cyberbullying on social networking sites: The deindividuation and moral disengagement perspectives. Information Systems Research. 2023;34(3):828–46. doi: 10.1287/isre.2022.1161

[35] Di Pentima L, Toni A, Roazzi A. Parenting styles and moral disengagement in young adults: The mediating role of attachment experiences. J Genet Psychol. 2023;184(5):322–38. doi: 10.1080/00221325.2023.2205451 37178171

[36] Maftei A, Burdea CA. Does perceived parental emotional warmth contribute to adults’ higher compassion? The mediating role of moral identity. Ethics & Behavior. 2023;34(7):506–21. doi: 10.1080/10508422.2023.2237148

[37] Hyde LW, Shaw DS, Moilanen KL. Developmental precursors of moral disengagement and the role of moral disengagement in the development of antisocial behavior. J Abnorm Child Psychol. 2010;38(2):197–209. doi: 10.1007/s10802-009-9358-5 19777337 PMC2858331

[38] Navas-Martínez MJ, Contreras L, Cano-Lozano MC. Mediating role of moral disengagement mechanisms in the relationship between perceived parental warmth and youth violence. Children (Basel). 2025;12(2):246. doi: 10.3390/children12020246 40003348 PMC11854850

[39] Di Pentima L, Toni A, Roazzi A. Moral development and parenting styles: The mediating role of emotional skills. Curr Psychol. 2024;43(18):16674–88. doi: 10.1007/s12144-023-05577-y

[40] Jiang Q, Yang Y-T, Liu C-L, Yuan J-W. The differing roles of cognitive empathy and affective empathy in the relationship between trait anger and aggressive behavior: A Chinese college students survey. J Interpers Violence. 2021;36(19–20):NP10937–57. doi: 10.1177/0886260519879229 31578910

[41] Bandura A. Selective moral disengagement in the exercise of moral agency. Journal of Moral Education. 2002;31(2):101–19. doi: 10.1080/0305724022014322

[42] Fang J, Wang X, Yuan K-H, Wen Z. Childhood psychological maltreatment and moral disengagement: A moderated mediation model of callous-unemotional traits and empathy. Personality and Individual Differences. 2020;157:109814. doi: 10.1016/j.paid.2020.109814

[43] Mateus Francisco S, Costa Ferreira P, Veiga Simão AM, Salgado Pereira N. Moral disengagement and empathy in cyberbullying: how they are related in reflection activities about a serious game. BMC Psychol. 2024;12(1):168. doi: 10.1186/s40359-024-01582-3 38515217 PMC10956178

[44] Chen H. Implicit weight stigma and bullying perpetration in college students: The mediating role of explicit weight stigma and moral disengagement and the moderating role of empathy. Psychology in the Schools. 2023;60(10):4143–58. doi: 10.1002/pits.22990

[45] Baltzidis E. Gender differences in cyberbullying perpetration on Facebook: the role of empathy, callous unemotional traits, and moral disengagement. Psychology, Society & Education. 2024;16: 53–62. doi: 10.21071/pse.v16i3.16997

[46] Barlett CP, Prot S, Anderson CA, Gentile DA. An empirical examination of the strength differential hypothesis in cyberbullying behavior. Psychology of Violence. 2017;7(1):22–32. doi: 10.1037/vio0000032

[47] Erdur-Baker O, Kavsut F. Cyber bullying: A new face of peer bullying. J Euroasian Educ Res. 2007;7:31–42.

[48] Zhou Z, Tang H, Tian Y, Wei H, Zhang F, Morrison CM. Cyberbullying and its risk factors among Chinese high school students. School Psychology International. 2013;34(6):630–47. doi: 10.1177/0143034313479692

[49] Matsunaga M. Item parceling in structural equation modeling: A primer. Communication Methods and Measures. 2008;2(4):260–93. doi: 10.1080/19312450802458935

[50] Jiang J, Lu Z, Jiang B, Xu Y. Revision of the short-form egna minnen av barndoms uppfostran for chinese. Psychol Dev Educ. 2010;26:94–9. doi: 10.16187/j.cnki.issn1001-4918.2010.01.017

[51] Wang X, Yang J. Reliability and validity of moral disengagement scale in Chinese students. Chin J Clin Psychol. 2010;18:177–9. doi: 10.16128/j.cnki.1005-3611.2010.02.025

[52] Jolliffe D, Farrington DP. Development and validation of the basic empathy scale. J Adolesc. 2006;29(4):589–611. doi: 10.1016/j.adolescence.2005.08.010 16198409

[53] Schoemann AM, Boulton AJ, Short SD. Determining power and sample size for simple and complex mediation models. Social Psychological and Personality Science. 2017;8(4):379–86. doi: 10.1177/1948550617715068

[54] Hayes, A. F. (2012). Process: A Versatile Computational Tool For Observed Variable Mediation, Moderation, And Conditional Process Modeling [White paper]. Retrieved from http://www.afhayes.com/public/process2012.pdf.

[55] Podsakoff PM, MacKenzie SB, Lee J-Y, Podsakoff NP. Common method biases in behavioral research: a critical review of the literature and recommended remedies. J Appl Psychol. 2003;88(5):879–903. doi: 10.1037/0021-9010.88.5.879 14516251

[56] Akinwande MO, Dikko HG, Samson A. Variance inflation factor: As a condition for the inclusion of suppressor variable(s) in regression analysis. OJS. 2015;05(07):754–67. doi: 10.4236/ojs.2015.57075

[57] Ryu E. Effects of skewness and kurtosis on normal-theory based maximum likelihood test statistic in multilevel structural equation modeling. Behav Res Methods. 2011;43(4):1066–74. doi: 10.3758/s13428-011-0115-7 21671139

[58] Li W, Zhang S, Lin H, Zhang K, Zhang X, Chen J, et al. Childhood maltreatment and creativity among Chinese college students: A serial mediation model. J Intell. 2023;11(4):58. doi: 10.3390/jintelligence11040058 37103243 PMC10147018

[59] Casselman RB, McKenzie MD. Young adults’ recollections of parental rejection and self-reported aggression: The mediating roles of insecure adult attachment and emotional dysregulation. Journ Child Adol Trauma. 2014;8(1):61–71. doi: 10.1007/s40653-014-0032-x

[60] Kahya Y, Akbaş D. Parental rejection as a developmental vulnerability factor for mood symptoms in young adulthood. J Child Fam Stud. 2025;34(2):543–59. doi: 10.1007/s10826-025-03008-z

[61] Kim M, Ellithorpe M, Burt SA. Anonymity and its role in digital aggression: A systematic review. Aggression and Violent Behavior. 2023;72:101856. doi: 10.1016/j.avb.2023.101856

[62] Zhou Q, Eisenberg N, Losoya SH, Fabes RA, Reiser M, Guthrie IK, et al. The relations of parental warmth and positive expressiveness to children’s empathy-related responding and social functioning: a longitudinal study. Child Dev. 2002;73(3):893–915. doi: 10.1111/1467-8624.00446 12038559

[63] Saleem S, Asghar A, Subhan S, Mahmood Z. Parental rejection and mental health problems in college students: Mediating role of interpersonal difficulties. PJPR. 2019;34(3):639–53. doi: 10.33824/pjpr.2019.34.3.35

[64] Ashburn R, Kazanas S. Parental childhood rejection: An exploration of anxiety and depression in later life. Modern Psychological Studies. 2024;30. Available: https://scholar.utc.edu/mps/vol30/iss1/1

[65] Marici M, Clipa O, Runcan R, Pîrghie L. Is Rejection, parental abandonment or neglect a trigger for higher perceived shame and guilt in adolescents?. Healthcare (Basel). 2023;11(12):1724. doi: 10.3390/healthcare11121724 37372842 PMC10298591

[66] Woszidlo A, Kunkel A. Social Learning Theory: An Emphasis on Modeling In Parent–Child Relationships. 2nd ed. ed. Routledge. 2017.

[67] Garaigordobil M. Psychometric properties of the cyberbullying test, a screening instrument to measure cybervictimization, cyberaggression, and cyberobservation. J Interpers Violence. 2017;32(23):3556–76. doi: 10.1177/0886260515600165 26289455

